# A cross-sectional study of experienced coercion in adolescent mental health inpatients

**DOI:** 10.1186/s12913-018-3208-5

**Published:** 2018-05-30

**Authors:** Olav Nyttingnes, Torleif Ruud, Reidun Norvoll, Jorun Rugkåsa, Ketil Hanssen-Bauer

**Affiliations:** 1 R&D department, Division of Mental Health Services, Akershus University Hospital, PB 1000, 1478 Lørenskog, Norway; 2 Institute of Clinical Medicine, University of Oslo, Oslo, Norway; 3 Work Research Institute, Oslo Metropolitan University, Oslo, Norway; 4 Health Services Research Unit, Akershus University Hospital, Oslo, Norway; 5 Centre for Care Research, University College of Southeast Norway, Notodden, Norway

**Keywords:** Adolescent psychiatry, Involuntary admission, Cross-sectional studies, Perceived coercion

## Abstract

**Background:**

Involuntary care and coercive measures are frequently present in mental healthcare for adolescents. The purpose of this study was to examine to what extent adolescents perceive or experience coercion during inpatient mental health care, and to examine predictors of experienced coercion.

**Methods:**

A cross-sectional sample of 96 adolescent inpatients from 10 Norwegian acute and combined (acute and sub-acute) psychiatric wards reported their experienced coercion on Coercion Ladder and the Experienced Coercion Scale in questionnaires. Staff reported use of formal coercion, diagnoses, and psychosocial functioning. We used two tailed t-tests and mixed effects models to analyze the impact from demographics, alliance with parents, use of formal coercion, diagnostic condition, and global psychosocial functioning.

**Results:**

High experienced coercion was reported by a third of all patients. In a mixed effects model, being under formal coercion (involuntary admission and / or coercive measures); a worse relationship between patient and parent; and lower psychosocial functioning, significantly predicted higher experienced coercion. Twenty-eight percent of the total sample of patients reported a lack of confidence and trust both in parents and staff.

**Conclusions:**

Roughly one third of patients in the sample reported high experienced coercion. Being under formal coercion was the strongest predictor. The average scores of experienced coercion in subgroups are comparable with adult scores in similar care situations. There was one exception: Adolescents with psychosis reported low experienced coercion and almost all of them were under voluntary care.

**Electronic supplementary material:**

The online version of this article (10.1186/s12913-018-3208-5) contains supplementary material, which is available to authorized users.

## Background

Coercion in mental health care remains controversial. Research is increasingly focused on the use, effect, and patient’s perception or experience of coercive treatment forms, but little is published on adolescent patients. Coercion is present in adolescent mental health care: in Norway, 20% of admissions among 16- to 17-year-olds was involuntary [1], although it was 36.5% in a German sample [2]. Adolescent inpatients may also be subjected to forced medication or nutrition for treatment purposes, as well as other coercive measures, such as holding, mechanical restraints, seclusion, and medication, in order to prevent harm to people or property. Some studies found that coercive measures are used more frequently for younger adolescents [3, 4]. The reported rate of inpatients subjected to one or more of these coercive measures ranged from 30% for inpatients in New York [5] and Finland [6] to 6.5% in Norway [7].

Other staff activities, such as inducements, interpersonal leverage, show of force, threats and house rules, can be experienced as coercive and regarded as informal pressure or informal coercion [8]. For adolescents, the magnitude of age, status, and knowledge differences vis a vis the staff can increase the influence of informal pressure. Furthermore, adolescents are usually materially, financially, and emotionally dependent on parents or guardians [9], so that control and pressure may relate to care, trust, and family loyalty. There is also a risk that some adolescent patients lack or lose trust in parents and staff during hospitalization, and consequently feel isolated in the ward. Coercion is often accompanied or followed by feeling rejected, aggrieved, punished [10] disempowered or terrified [11]. Some prospective studies have connected experienced coercion to lower quality of life [12], and worse alliance and follow up of care [13], but a review found small or absent effects on variables such as psychosocial functioning, readmissions, or service engagement [14]. Given frequent use of formal coercion, the potential for informal pressure or coercion, and the vulnerable adolescent years, experienced coercion in adolescents should be an important research topic [15].

A review of adolescent experiences with mental health care found few inpatient studies, and the main topic was patient satisfaction [16]. One recent American interview study with inpatients found that rigidity and confinement were the most frequently disliked aspects of care [17]. A few interview studies have reported how adolescent and young adult inpatients with anorexia view treatment: Patients are aware of staff strategies for influence, such as persuasion and use of patient privileges. Some patients attempt to resist or circumvent treatment, i.e., some play by the rules to get out, and some attenuate staff authority by questioning their competence [18]. Patients spoke about formal coercion and informal pressure, with some saying that coercion and restrictions could at times be justified and helpful [19]. In one study, adolescents with eating disorders reported more experienced coercion than adults [20].

The literature on coercion in adults is far more extensive, with subfields such as outpatient coercion with community treatment orders, coercive measures, and perceived or experienced coercion. In 1993, the development of the Admission Experience Survey and its subscale the MacArthur Perceived Coercion Scale [21] stimulated a series of studies on perceived coercion. These studies found that involuntary care predicts perceived coercion, although approximately 35% of involuntary patients in acute wards reported low perceived coercion in several studies [22, 23]. Conversely, the number of voluntarily admitted patients who reported a high perceived coercion score, ranged from 10% in the original MacArthur studies [24] to 48% in a smaller English study [25]. Across studies, the odds ratio of patients under involuntary care reporting high perceived coercion compared to voluntary patients was 8.6 [26]. Use of physical force or threats of social consequences for treatment also predicts higher perceived coercion in patients [14]. A higher level of perceived procedural justice – i.e., feeling that you had a say in the decision and considered the admission process to be fair – are associated with lower perceived coercion [27]. Also, a positive relation to the clinician is associated with lower perceived coercion [25]. Research on the impact of demographic and clinical characteristics displayed small and inconclusive effects [28]. Thus, we lack a clear and documented understanding of the interrelation between the main explanatory variables of perceived coercion, such as patient characteristics, care regimen, alliance, and procedural justice. Qualitative studies indicate that patients do not equate freedom restrictions to perceived coercion, but restricts the coercion concept to negatively viewed restrictions, such as the humiliating ones [29]. In addition, they described coercion as a broader experience affecting self-image, and sometimes with existential consequences [30, 31]. For patients, coercion seems to be more of a negative experience than merely a perception, making experienced coercion the preferred concept.

Our main study aims were to establish the level of experienced coercion and test candidate predictor variables in a sample of hospitalized adolescents. Based on existing findings for adults [32, 33] and how formal coercion is used for adolescents [1, 5] we hypothesized that younger age, use of formal coercion (involuntary care, coercive treatment or measures), eating disorders, and lower global psychosocial functioning would predict higher experienced coercion. However, as eating disorders [1] and lower psychosocial functioning [34] are associated with increased use of formal coercion, we expected these clinical variables to lose significance when controlled for use of formal coercion. Additional study aims were to explore:

- Whether a good relation to the parent or guardian would predict higher or lower experienced coercion;
- Whether pressure for admission from parents would have different effects on experienced coercion for voluntary vs involuntary patients;
- What proportion of patients would report lack of trust or closeness towards both parents and staff.

For voluntary patients, we expected that pressure from parents (re: admission) would lead to higher experienced coercion compared to patients without such pressure. However, for involuntary patients, such pressure could be insufficient to add to experienced coercion, and might contribute to a sense of necessity and legitimate care, with less experienced coercion.

## Methods

### The study context

In Norway, per 100,000 underage persons (aged 0–17), there are 26 mental health inpatient beds used yearly by 180 patients in 249 admissions [35]. The adolescent wards in this study accept patients from 13 to 17 years. This age group uses approximately 75% of the total underage inpatient capacity [36], indicating that in 2014, 0.5% of the adolescent population received inpatient mental health care. Adolescent inpatients in Norway are thus a highly select group, expected to have severe mental health problems, which services consider difficult to administer proper care in outpatient settings. Norwegian adolescent acute and sub-acute units are small but well-staffed, usually with 10 or fewer beds per ward, and with staffing (including administrative) of more than 4 employees per bed [35].

According to the Norwegian Mental Health Act, patients 16 and above can be involuntarily admitted and treated according to the same rules as adults. Patients less than 16 are admitted based on parental consent, and are thus formally seen as voluntary [37]. The ward shall notify the Control Commission (a tribunal board for complaints about involuntary mental health care) whenever an admitted patient under 16 disagrees with the parents’ decision.

### Design

We conducted a cross-sectional study of adolescent inpatients from 10 Norwegian acute and combined acute and sub-acute psychiatric wards. Data were collected from patients, staff, and clinical records.

### Recruitment of wards

We sent an invitation to participate to all 16 Norwegian adolescent wards (acute and combined acute and intermediate inpatient) approved for involuntary care. Ten out of these wards participated in the study.

### Patient inclusion and data collection

Data collection took place in 2015. Each participating ward chose a start-up day for recruitment. At this point, all admitted patients in the ward regardless of care formality, were considered for eligibility. Patients’ inclusion criteria were being 13- to 17-years-old, competency to consent by understanding the consequences of participating, and the ability to comprehend a two-page questionnaire. Patients were approached by local clinicians, who gave them information about the study and requested consent to participate. For patients under the age of 16, parents were also asked for consent. The patient was asked to fill out a form with questions and statements (see Additional file 1), preferably in private, and to enclose it in an envelope themselves. Staff assisted with reading or explanations if needed. The patient’s primary contact or responsible clinician also filled out a form about the patient and treatment based on the patient’s record and past care (see Additional file 2). Recruitment procedures were repeated weekly for newly-admitted patients until the ward reached its goal, based on ward size, or gave up recruitment.

### Measurements

We used paper forms filled out by patients and therapists to measure the variables selected for this study. Members of the adolescent group of the Norwegian Acute Psychiatric Network suggested clinically-relevant variables as well as their wording.

#### Experienced coercion

No measure of experienced coercion has been validated for adolescents, so we chose two measures developed for adults with complementary strengths, and we report and compare both. *The Coercion Ladder* (CL) is a one-item, self-anchoring visual analogue scale based on the Cantril Ladder [38], measuring one’s recent experience of being coerced. The score range is 1–10 and the respondents are instructed that the lowest and highest scores should correspond to the lowest and highest level of experienced coercion they can imagine. The participant’s understanding of the word ‘coercion’ is the anchor. This may sacrifice reliability, as found in other iterations of Cantril’s approach [39], but should be directly applicable to adolescent mental health care and adolescents’ understanding of the word ‘coercion.’ *The Experienced Coercion Scale* (ECS) has 15 agreement-rated five-point Likert items, and the score range is 0–4. Items are applicable across care phases, care settings, and forms of coercion, focusing on patients’ negative evaluations and feelings [33]. We calculated average sumscore from valid item responses. For both scales, we defined high experienced coercion as a score above the midpoints [> 5 on CL, > 2 on the ECS). Patients also noted if they *agreed with the admission* and if they thought their parents or other parties agreed with it too.

#### Use of formal coercion

Involuntary admission was coded ‘yes’ if the adolescent was 16 to 17 years old, and involuntary admitted according to clinical records. This variable was also coded ‘yes’ for younger patients who disagreed to being admitted, warranting a notification to the Control Commission. Data about *coercive measures,* such as involuntary medication, involuntary nutrition, restraints, and open door seclusion, which happened during the last three weeks of admission, was reported by staff. *Patients under formal coercion* were those who had experienced any involuntary admission or coercive measure described in this paragraph.

#### Clinical status

*Diagnosis* was measured as the main psychiatric disorder using Axis One (clinical psychiatric syndromes) in the multiaxial ICD-10 Classification of Child and Adolescent Psychiatric Disorders from the World Health Organization [40]. This was found in the patient’s record during data collection. *Global psychosocial functioning* was measured using the units’ routine application of the Children’s Global Assessment Scale (CGAS) [41] at admission, and by asking the clinician to rate the CGAS at the time of the patient’s response. Staff rated the patient with Health of the Nation Outcome Scales – Children and Adolescents (HoNOSCA) for *use of alcohol or drugs* [42] in the last 6 months. HoNOSCA defines non-problematic use as no use or use within age norms. *Length of stay* and the *living situation from which the adolescent was admitted* were rated by staff using the patient records.

#### Relation between patient and parent/guardian

The *quality of the patient’s relation to parent* and *staff* was measured with a set of agreement-rated Likert-items. In this section, we rewrote and adapted the expectation of help from mother/father in the Conflict Behaviour Questionnaire [43]. The patient rated parent and staff on separate items. We were similarly informed of the theme of openness and trust from the Scale to Assess the Therapeutic Relationship [44], and coined an item of hiding inner feelings, which the patient rated for parents and staff. Also, staff rated the relation between patient and parent or guardian. We calculated a combined measure of *patients’ relation towards the parent* as the average score on two patient-rated items and one staff-rated item*,* where higher scores indicated better relations*.* An item was added after the pilot interviews, acknowledging the nuance between hiding one’s inner feelings from the parent due to lack of trust or in order to spare them from knowing convoluted feelings or situations. Staff rated the degree of *informal pressure from parents* on a self-made 5-point Likert item.

*Gender* was marked by the patients, and *age* was reported by the staff. Ethnic or immigrant backgrounds were not recorded.

We piloted the patient questionnaire with a cognitive validation interview [45], with three patients at two sites, and inquired how items were understood, how the patient reasoned, and how he/she thought other patients would reason when answering the form. Pilot interviews indicated that patient items, including experienced coercion scales, were understood.

### Study sample

**Fig. 1:**
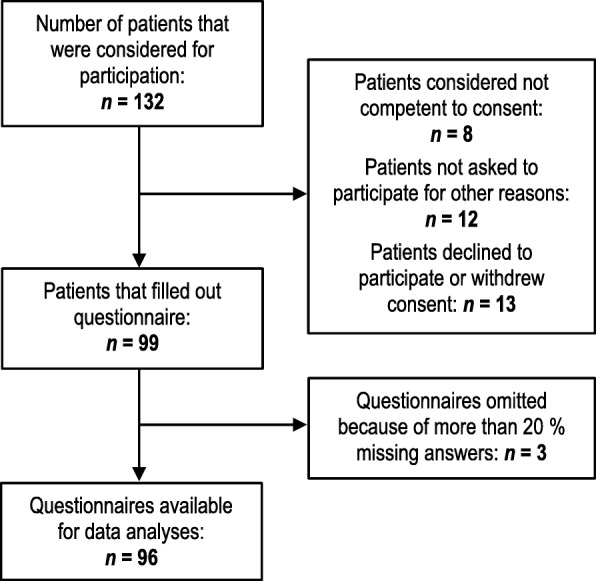
Flow chart of recruitment, exclusions, and refusals of patients

Among 132 patients considered for participation, data from 96 (73%) were included in the analyses, as shown in Fig. 1. We excluded three cases with more than 20% missing ECS items. For remaining participants, CL had no missing data. ECS items had a total of 15 missing answers (1.04%). No participant missed more than two item responses on the ECS.

### Statistical analyses

We analysed data with SPSS 23. CL scores showed a skewed distribution, so we utilized Spearman’s rank order for correlation with this scale. Parametric tests were restricted to ECS scores, in which we studied the predictive value of use of formal coercion and diagnosis with two tailed t-tests, Cohen’s *d* effect size, and ANOVA. We used ECS sum scores as the dependent variable and estimated a linear mixed effects model. Here we entered age, relation to parent or guardian, global psychosocial functioning, eating disorders, and use of formal coercion as fixed effects and estimated a random intercept for the effect of wards. Non-dichotomous predictors were centered at their grand mean. In a second model, we explored whether informal pressure for admission from the parents influenced experienced coercion differently for voluntary patients compared to patients under formal coercion, using an interaction variable.

## Results

The sampling procedure resulted in adolescent inpatients with characteristics shown in Table 1. More girls (68.8%) than boys and more older (65.6%) than younger adolescents participated. Staff reported that 81 (86.2%) patients had non-problematic use of alcohol and drugs.

**Table 1:** Patient characteristics

	13–15 years *n = 33*	16–17 years *n = 63*	Total *n = 96*
	n (%)	n (%)	n (%)
*Gender*			
Female	22 (66.7)	44 (69.8)	66 (68.8)
Male	11 (33.3)	19 (30.2)	30 (31.3)
*Diagnosis (ICD-10 codes)*			
Psychosis (F20–31)	9 (27.3)	5 (7.9)	14 (14.6)
Pervasive developmental disorder (F84)	1 (3)	6 (9.5)	7 (7.3)
Eating disorders (F50)	5 (15.2)	9 (14.3)	14 (14.6)
Depressive disorder (F32–34)	9 (27.3)	20 (31.7)	29 (30.2)
Anxiety, dissociative disorders, PTSD (F40–44; F92–94)	5 (15.2)	9 (14.3)	14 (14.6)
All other disorders^a^ (incl. missing)	4 (12.1)	14 (22.2)	18 (18.8)
*Living situation before admission*			
Living at home	26 (78.8)	50 (79.4)	76 (79.2)
Living in institution or foster care	6 (18.2)	9 (14.3)	15 (15.6)
Not specified (other or missing)	1 (3)	4 (6.3)	5 (5.2)
*Length of stay at the time of data collection*			
Short (1–4 days)	8 (24.2)	23 (36.5)	31 (32.3)
Medium (5–21 days)	17 (51.5)	21 (33.3)	38 (39.6)
Long (22 days or longer)	8 (24.2)	15 (23.8)	23 (24)
Missing		4 (6.3)	4 (4.2)
*Involuntary admission*			
No	29 (87.9)	49 (77.8)	78 (81.3)
Yes	4 (12.1)	14 (22.2)	18 (18.8)
*Episode of coercive measure* ^b^ *for last three weeks*			
No	32 (97)	54 (85.7)	86 (89.6)
Yes	1 (3)	8 (12.7)	9 (9.4)
Missing	0 (0)	1 (1.6)	1 (1)
*Children’s Global Assessment Scale*	mean (sd)	mean (sd)	mean (sd)
At admission^c^	38.5 (8.8)	35.7 (13.8)	36.7 (12.3)
At time of data collection^d^	44.6 (9.1)	40.7 (13.8)	42.1 (12.5)

ICD-10: International Statistical Classification of Diseases and Related Health Problems, 10th Revision

*sd* standard deviation

^a^Personality disorder (F60), Hyperkinetic disorder (F90), Unspecified mental disorder (F99), Auditory hallucinations (R44.0), Suicidal ideation (R45.8), Observation for suspected mental or behavioral disorder (Z032)

^b^Coercive measures could include physical holding, mechanical restraints, medication, nutrition, isolation, or open door seclusion

^c^Missing data for 3 patients

^d^Missing data for 13 patients

### Experienced coercion among adolescent inpatients

**Fig. 2:**
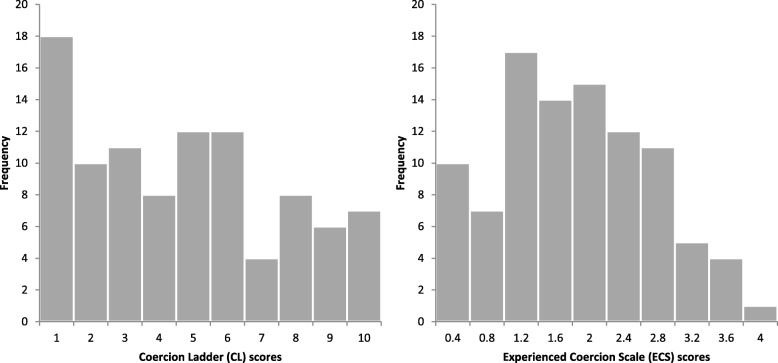
Histogram of Coercion Ladder scores and Experienced Coercion Scale (ECS) average sumscores in the sample. *N* = 96

The patients’ mean score on CL was 4.7 (SD = 2.9, median score = 5). The mean score for patients under formal coercion was 7.3 (SD = 2.6, median score = 8) while voluntary patients’ mean score was 4.1 (SD = 2.6, median score = 4). The mean score on the ECS (scaled from 0 to 4) was 1.7 (SD = 0.9). The correlation between CL scores and the ECS sum scores was *r*_s_ = .68. The distribution of both scales of experienced coercion is shown in Fig. 2.

The mean difference in experienced coercion as measured by the ECS, between patients under formal coercion (2.4 points, *n* = 19) and the non-coerced patients (1.5 points, *n* = 77) was 0.9 points [0.5, 1.3], with t (94) = 4.16, *p* < .001, *d* = 1.01.

In the total sample, 33 of the patients (34.4%) reported high experienced coercion (ECS score > 2). For adolescents under formal coercion, the percentage was 73.7, while 24.7% of the voluntary patients reported high experienced coercion.

### Predictors of experienced coercion

Among the 96 patients, 46 (47.9%) agreed that they ought to be treated on the ward. Of these, 12 patients (26.1%) nevertheless reported high experienced coercion on the ECS. Fifty patients did not agree with treatment on the ward, and 28 of these (56.0%) reported low experienced coercion according to the ECS. A majority of 62 patients (64.6%) thought their parents endorsed the current stay. Only 16 patients (16.7%) disagreed with treatment on the ward *and* thought the parents did not endorse the current stay. Here, the adolescent considered Child Protection Services (5 patients (5.2%)) and Child and Adolescent Mental Health Care (10 patients (10.4%)) as proponents of their current treatment.

We found significantly different levels of experienced coercion for patients in the diagnostic groups shown in Table 1, as implied by ANOVA with *F*(5,90) = 2.570, *p* = .032. A Tukey post hoc test revealed that the experienced coercion score of 2.29 in patients with eating disorders was significantly higher compared to that of 1.20 in patients with psychosis (*p* = .016). Other differences were nonsignificant. Eight of 14 patients with eating disorders, but only 1 of 14 patients with psychosis were under formal coercion.

In the first step of multilevel modeling, we estimated a model not including any predictors, but accounting for the variation in experienced coercion between wards. The intraclass correlation (ICC) was 0.072. Akaikes information criterion was 225.443 for this model. Then we added the predictors shown in Table 2 as fixed effect variables in the model. In this model, ICC for ward was 0.102, and the Akaike information criterion was 208.456, indicating a smaller information loss when we included the predictors. Being under formal coercion, having lower psychosocial functioning and a worse relation to parents or guardian were significant predictors of higher experienced coercion score in the model. Age and eating disorders were non-significant. Parameter estimates with confidence intervals (CIs) are in Table 2.

**Table 2:** Parameter estimates of predictors of Experienced Coercion Scale scores with a random intercept for wards in a mixed effects model

Parameter	Estimate	95% CI
Fixed		
Intercept	1.466**	[1.163, 1.769]
Patient age^a^	− 0.114	[− 0.257, 0.028]
Patients’ relation to parent or guardian^a^	− 0.258*	[−0.425, − 0.091]
Global psychosocial functioning (CGAS)^a^	− 0.021*	[−0.039, − 0.003]
Diagnosed with eating disorder (reference: no)	0.341	[−0.158, 0.840]
Patient under formal coercion (reference: no coercion)	0.805**	[0.353, 1.257]
Covariance		
Residual standard deviation	0.546	[0.393, 0.798]
Between wards standard deviation	0.062	[0.009, 0.450]

*CI* confidence interval

^a^Non-dichotomous variables are grand mean centered

* *p* ≤ .05. ** *p* ≤ .001

To assess if informal pressure from the parents influenced experienced coercion differently in voluntary and coerced patients, we estimated a second mixed effects model with fever parameters but adding interaction between informal pressure from parents and being under formal coercion. In this model, ICC for ward was 0.088 and the Akaike information criterion was 231.895. Removing the interaction parameter increased the Akaike information criterion to 234.536. The parameter estimates are given in Table 3.

**Table 3:** Parameter estimates for effect of parent or guardian pressure on Experienced Coercion Scale scores with a random intercept for wards in a mixed effects model

Variable	Estimate	95% CI
Fixed		
Intercept	1.543**	[1.281, 1.805]
Patients’ relation to parent or guardian^a^	−0.232*	[− 0.392, − 0.072]
Informal pressure from parent or guardian^a^	0.222*	[0.059, 0.385]
Patient under formal coercion (reference: no coercion)	0.902**	[0.489, 1.314]
Informal pressure from parent or guardian x patient under formal coercion	−0.358*	[− 0.697, − 0.019]
Covariance		
Residual standard deviation	0.573	[0.422, 0.777]
Between wards standard deviation	0.056	[0.009, 0.3570]

*CI* confidence interval

^a^Non-dichotomous variables are grand mean centered

* *p* ≤ .05. ** *p* ≤ .001

The analyses show that voluntary patients rate higher experienced coercion when there is more informal pressure from parents or guardians. For patients under formal coercion, more informal pressure predicted *lower* experienced coercion.

### The patients’ trust in parents and staff

Fifty-six (58.9%) patients agreed or strongly agreed they would not show their parents how they really felt, either due to lack of trust (14 patients) or to spare the parents (19 patients), or for both reasons (23 patients). Thirty-seven patients (38.5%) would not show staff how they felt. Twenty-six patients (27%) would not show how they felt to either parents or staff.

## Discussion

To our knowledge, this is the first quantitative study of experienced coercion in adolescent mental health care. The study adds valuable knowledge regarding degree and predictors of experienced coercion.

The level of experienced coercion, as measured by the ECS and the CL, was in a similar range as in reports from adult samples. On the ECS, adolescents under formal coercion scored 2.4 points, while patients under involuntary care in a Norwegian adult sample scored 2.2 points [33]. The scores for voluntary patients were 1.5 and 1.3 points in the adolescent and adult samples, respectively. The correlation between CL and the ECS was in the same range in this study as in the ECS validation study, with *r*_s_ = .68 in both studies. About 1/3 of the sample, and 3/4 of the patients under formal coercion, reported high experienced coercion. Adolescent inpatient stays may be formative for future alliance, concordance with care plans, and possibly influence later treatment results. We therefore see a need for prospective studies examining the consequences of experienced coercion in adolescent mental health care.

Experienced coercion varied with diagnosis. As expected, patients with eating disorders reported higher experienced coercion. Surprisingly, patients with psychosis reported low experienced coercion, and only one of these patients was involuntary admitted or subjected to coercive measures. Psychosis and psychotic symptoms have repeatedly been connected to more coercion and higher experienced coercion in adult samples [32, 46]. In Norway, 62% of all adult involuntary inpatient time was for patients with a main diagnosis of schizophrenia [47]. Nevertheless, Norwegian adolescent inpatient wards seemed able to care for most psychotic patients without formal involuntary care or experienced coercion for them. More studies are needed to rule out bias in our results, and to investigate how non-coercive psychosis care is accomplished.

Although the strongest predictor of experienced coercion was being under formal coercion, approximately ¼ of patients under formal coercion reported low experienced coercion, and approximately ¼ of voluntary patients reported high experienced coercion. Another significant predictor in the mixed effects model was negative relations with parents, which may stem from more relational problems in general. Age did not predict experienced coercion, and this hypothesis was based on findings of more frequent use of coercive measures for younger patients [1]. In Table 1, we see no sign of such a tendency in this sample. Patients with eating disorders reported high experienced coercion, but this may have been mediated by being under formal coercion, making eating disorders insignificant in the mixed effects model. The explanatory power of patient characteristics varies between existing studies of experienced coercion. Our results indicate that sometimes a more restrictive care regimen may mediate the effect of patient variables on experienced coercion. For some variables, there may be competing causal chains at work. The mixed effects model in Table 2, indicates that a worse psychosocial functioning predicted higher experienced coercion. Better global psychosocial functioning may protect from some care restrictions, leading to lower experienced coercion. However, better psychosocial functioning indicates that involuntary admission is less proportional, which may lead to less acceptance of the admission, as found in an English study [48]. In the former case a thorough multivariate control for all care restrictions should remove the significance of psychosocial functioning. A main effect cannot be ruled out either, in which lower psychosocial functioning may weaken a patient’s ability to see the care situation from different perspectives, creating a sense of more experienced coercion in an otherwise comparable care situation. While studies may control for formal coercion, it is difficult to rule out that effects of patient variables are mediated by informal pressure or coercion. In order to resolve these questions, a validated measure of informal pressure and restrictions in care, preferably reported by sources other than the patient, would seem to be necessary.

Given adolescent dependency on parents, how does informal pressure from caregivers predict experienced coercion? Our post hoc mixed effects model shows that pressure from parents predicted higher experienced coercion on the ward for voluntary patients. But for patients under formal coercion, informal pressure from parents was associated with lower experienced coercion, although the subsamples were small in this model. We speculate that this effect may be due to the parental legitimization of the involuntary care.

How did inpatients assess their alliance and trust in staff and parents? Almost half the patients agreed to treatment in the ward. Nevertheless, 27% of inpatients did neither report a good alliance with their parents nor the staff. The study sample is a highly select group based on problem severity. Lack of trust in adult relations may be a part of the situation for several adolescent inpatients. This may contribute to their problems, and make them particularly lonely and vulnerable. If, for some reason, understanding, empathy, or care quality breaks down, then the staff, the control system, or the parents cannot rest assured that an adolescent will discuss it with a parent. As implied in the pilot interviews, some adolescents may hide their negative feelings and experiences from parents to spare them a burden. This may be the case if parents initiated or agreed with admission, and if the alliance or treatment results eventually soured.

### Limitations

The study sample is small, partly reflecting the small adolescent wards. This sample size implies that findings on subgroups should be treated with caution. On the other hand, the rate of missing data was low from both patients and staff. Ten out of 16 Norwegian adolescent acute wards participated, and the participation rate on the wards was high. ICC for wards explained less than 10% of the variation in experienced coercion. Also, we received no reports of problems from the involved clinicians, such that the adolescents seem to have handled the questionnaire well.

Another limitation to this study is that the scales of experienced coercion have not previously been applied or validated in adolescent populations. We did not use the frequently-used MacArthur Perceived Coercion Scale, as it was developed and validated for an *adult admission process*, with little regard for parent authority and involvement. We piloted the patient form, and included two measures of experienced coercion. The correlation between these two measures was *r*_s_ = .68, as for adults. This similarity between the self-anchoring CL and ECS with items of negative valence indicates that adolescents delimit the coercion concept to freedom restrictions that are experienced negatively. Scale revisions or separate development for adolescents is preferable, however. Some other variables were also measured with items adapted or developed for this study, which have not yet been validated. For the patient-parent relation we combined two patient-reported items and one staff-reported item, and attributes of adolescents and families with a certain rating is not known. In particular, the parent perspective is not included, and the findings must be considered as tentative. Diagnosis and CGAS was based on clinicians’ assessments, and not tested for reliability. These clinical variables reflect the staff considerations and perspective, and include the breadth of the assessment, supplemental information and observation. While the diagnosis should have a broad base and be informed by the cooperation between professionals on the ward and cooperating services, the CGAS-score practice may vary more from one ward to another.

Generalizability is limited by the sample size and the study context. The organisation of mental health services for children and adolescents shows great variation across countries [49]. In Norway, the proportion of underage persons in contact with the outpatient division of Child and Adolescent Mental Health Services was 5.1 in 2014 [35]. From 1998 to 2013, around 0.03% of underage persons were hospitalised each year [36], and for Norwegian adolescent’s about 0.5% received a mental health inpatient stay in 2014.

The sample had a majority of girls (69%), close to the yearly national rates (65%) [35]. Severe diagnoses, such as psychosis, eating disorders, and pervasive developmental disorders made up 36.5% of this sample, while national all-year statistics for 2014 indicates that these disorders amounted to 21% [35]. Our sampling was cross-sectional, and patients with more severe problems often have longer stays and a greater likelihood for sampling than those with shorter stays. We think the reason for a low rate of externalizing behavioural disorders is that inpatient care for this group is often mandated by the Norwegian Child Protection Services.

## Conclusions

The level of experienced coercion in adolescent inpatient care found in this study was similar to comparable results for adult inpatient care. Use of formal coercion is the strongest predictor of experienced coercion, so use of coercion in adolescent mental health care should receive similar attention as in research and policies for adults. Norwegian adolescent wards treated psychosis with little use of formal coercion, and these patients also reported low experienced coercion.

## Additional files

Additional file 1: Patient report form. An English translation of the patient report form utilized in the study. (PDF 16 kb)

Additional file 2: Staff report form. An English translation of the staff report form utilized in the study. (PDF 23 kb)

## Abbreviations

CGAS: Children’s Global Assessment Scale
CL: Coercion Ladder
ECS: Experienced Coercion Scale
HoNOSCA: Health of the Nation Outcome Scales – Children and Adolescents
ICC: Intraclass correlation
ICD-10: International Statistical Classification of Diseases and Related Health Problems, 10th Revision
