# Supplementary material for: A cross-sectional study of experienced coercion in adolescent mental health inpatients
Source: BMC Health Serv Res. 2018 May 30;18:389. doi: 10.1186/s12913-018-3208-5 (PMC5977498; doi:10.1186/s12913-018-3208-5)
Supplement: Supplementary file 1 — Patient report form. An English translation of the patient report form utilized in the study. (PDF 16 kb) [file 12913_2018_3208_MOESM1_ESM.pdf]

Questionnaire regarding experienced coercion at the CAMH-unit

Please answer these questions about yourself first:

1 **Sex:** ☐ Girl ☐ Boy

2 **Age:** ☐ 13-15 years ☐ 16-18 years ☐ over 18 years

3 Who thinks that you should have this treatment at the CAMH-unit
(Mark all alternatives that you think apply)

☐ Yourself
☐ Parent / Guardian
☐ Other family
☐ A friend
☐ School / teachers
☐ Child protection agency
☐ Police
☐ Medical emergency unit
☐ GP
☐ The CAMH-unit

Others (if applicable):

		Strongly disagree	Disagree	Neiter agree nor disagree	Agree	Strongly agree
4	When I really need something, it is helpful to turn to one of my parents/guardians	☐	☐	☐	☐	☐
5	I do not trust my parents / guardians, and do not show them how I really feel inside	☐	☐	☐	☐	☐
6	I try to spare my parents / guardians, and do not show them how I really feel inside	☐	☐	☐	☐	☐
7	When I really need something, it is helpful to turn to the primary contact or responsible clinician	☐	☐	☐	☐	☐
8	I prefer not to show the primary contact or responsible clinician how I really feel inside	☐	☐	☐	☐	☐

9 **How coerced do you currently feel at the CAMH-unit?**
Please mark the step of the ladder that fits best
Put a mark on the step that best fits the care that you currently receive

10 ☐ The highest possible level of experienced coercion
9 ☐
8 ☐
7 ☐
6 ☐
5 ☐
4 ☐
3 ☐
2 ☐
1 ☐ The lowest possible level of experienced coercion

In addition, a form with the Experienced Coercion Scale was printed on the return page of the form. This scale is presented in Nyttingnes, O., Holmén, A., Rugkåsa, J., & Ruud, T. (2016). The Development, Validation, and Feasibility of the Experienced Coercion Scale (ECS). Psychological Assessment, Advance online publication. Retrieved from doi:dx.doi.org/10.1037/pas0000404.
