# Supplementary material for: A cross-sectional study of experienced coercion in adolescent mental health inpatients
Source: BMC Health Serv Res. 2018 May 30;18:389. doi: 10.1186/s12913-018-3208-5 (PMC5977498; doi:10.1186/s12913-018-3208-5)
Supplement: Supplementary file 2 — Staff report form. An English translation of the staff report form utilized in the study. (PDF 23 kb) [file 12913_2018_3208_MOESM2_ESM.pdf]

Experienced coercion - Staff information sheet

Fill in the form after the patient / patient and parent has consented and filled in their form. When not otherwise specified, think of the situation on the day the patient completed his / her form.

A: Information regarding patient and admission

1 **Age of the patient:** ☐ **Was the patient 16 years or more at admission:** ☐ yes ☐ no

2 **ICD-10-diagnoses (in code form)**

	Axis I	Axis II	Axis III	Axis IV	Axis V
Major diagnosis:	☐	☐	☐	☐	☐
Supplementary diagnoses:	☐	☐		☐	☐

3 **Functioning: CGAS at admission:** ☐ **CGAS (scored based on the last three days):** ☐

4 **Living arrangement immediately before the current admission**
☐ in the home ☐ in institution. (CPS, MHC, prison etc) ☐ in foster home ☐ absconded / vagabonding ☐ emergency home ☐ other

5 **Duration of the current admission:** ☐ 1-4 days ☐ 5-21 days ☐ more than 3 weeks

6 **Presence at school or work the last 4 weeks before admission (home education do not count as presence).** In case of single lesson absence, let 4 single hours absence count as 1 day absence, 8 hours as 2 days etc.
- ☐ **low absence** (17 to 20 days presence / 0-19% absence)
- ☐ **moderate absence** (11 to 16 days presence / 20-49% absence)
- ☐ **high absence** (5 to 10 days presence / 50-79% absence)
- ☐ **very high absence** (0 to 4 days presence / 80-100% absence)
- ☐ **not applicable (for example not offered schooling)**
- ☐ **school presence unknown**

7 **Problems with alcohol, substance / solvent misuse, taking into account current age and societal norms:**
- ☐ **no use, or minor use** within age norms
- ☐ **mildly** exessive alcohol or drug use
- ☐ **moderately** severe drug or alcohol problems significantly out of keeping with age norms
- ☐ **severe** drug or alcohol problems leading to dependency or incapacity
- ☐ **lack of information / don't know**

B: Use of coercion according to the Norwegian Mental Health Act

8 The **current** treatment formality in the Mental Health Act:
☐ § 2-1 (consent) ☐ § 2-2 (consent to be held back) ☐ § 3-2 (involuntary observation) ☐ § 3-3 (involuntary care)

9 The treatment formality at **referral and at the beginning of the current stay:**
Treatment formality at the beginning of admission: ☐ § 2-1 ☐ § 2-2 ☐ § 3-2 ☐ § 3-3
Referral asked for coercion ☐ yes ☐ no

10 Did the admission qualify for notifying the control commission about **parental consent against the adolescent will**?
☐ yes ☐ no ☐ not applicable, the patient was 16 or more at the point of admission

11 Is the patient under involuntary treatment measures according to §4-4 (more than one form may apply to each patient):
☐ No ☐ § 4-4a (medication) ☐ § 4-4b (nutrition) ☐ Other involuntary treatment measures

12 Has the patient been subjected to coercive measures during the last 3 weeks? (include restraints during nutrition)
☐ no ☐ yes, § 4-8a (mech. restr.) ☐ § 4-8b (isolation) ☐ § 4-8c (fast acting sedatives) ☐ § 4-8d (phys. restr.)

13 Has the patient been shielded / open door secluded according to §4-3 in the last 3 weeks ☐ no ☐ less than one day
Shielding notified in the coercion protocol: ☐ up to one day ☐ shielded at 2-4 days ☐ shielded at 5 or more days

C: Information, as evaluated by the primary contact or responsible clinician:

	**To what degree do the following apply to the patient or describe the current situation?** Base the marks on an overall evaluation	No degree	Small degree	Some degree	Strong degree	Very strong degree
14	The patient thinks he / she needs help	☐	☐	☐	☐	☐
15	The patient has a good relation to at least one of his / her parents / guardians	☐	☐	☐	☐	☐
16	The patient has a good relationship to the ward staff	☐	☐	☐	☐	☐
17	The parents / guardians have pressured the patient to accept treatment	☐	☐	☐	☐	☐
18	Staff has pressured the patient to accept treatment	☐	☐	☐	☐	☐
19	The patient easily feels violated	☐	☐	☐	☐	☐
20	The patient easily feels opposed	☐	☐	☐	☐	☐
